# The research environment of critical care in three Asian countries: A cross-sectional questionnaire survey

**DOI:** 10.3389/fmed.2022.975750

**Published:** 2022-09-20

**Authors:** Yuki Kotani, Sungwon Na, Jason Phua, Nobuaki Shime, Tatsuya Kawasaki, Hideto Yasuda, Jong Hun Jun, Atsushi Kawaguchi

**Affiliations:** ^1^Department of Intensive Care Medicine, Kameda Medical Center, Kamogawa, Japan; ^2^Department of Anesthesiology and Pain Medicine, Anaesthesia and Pain Research Institute, Yonsei University College of Medicine, Seoul, South Korea; ^3^FAST and Chronic Programmes, Alexandra Hospital, National University Health System, Singapore, Singapore; ^4^Division of Respiratory and Critical Care Medicine, Department of Medicine, National University Hospital, National University Health System, Singapore, Singapore; ^5^Department of Medicine, Yong Loo Lin School of Medicine, National University of Singapore, Singapore, Singapore; ^6^Department of Emergency and Critical Care Medicine, Graduate School of Biomedical and Health Sciences, Hiroshima University Hospital, Hiroshima, Japan; ^7^Department of Pediatric Critical Care, Shizuoka Children's Hospital, Shizuoka, Japan; ^8^Department of Emergency and Critical Care Medicine, Jichi Medical University Saitama Medical Center, Saitama, Japan; ^9^Department of Anesthesiology and Pain Medicine, Hanyang University College of Medicine, Seoul, South Korea; ^10^School of Medicine, Department of Pediatrics, St. Marianna University, Kawasaki, Japan; ^11^CHU Sainte Justine Research Centre, University of Montreal, CHU Sainte Justine Research Centre, Montreal, QC, Canada

**Keywords:** research activities, cross-sectional studies (MeSH), community hospital, Asia, critical care

## Abstract

Although inadequate research support for intensivists can be one major reason of the poor research productivity, no study has investigated the current research environment in critical care medicine in Asia. The objective of this study was to describe Asian academia in critical care from the research environment perspective. We conducted a cross-sectional questionnaire survey targeting all physician members of the Societies of Intensive/Critical Care Medicine in Japan, South Korea, and Singapore. We collected the characteristics of the participants and their affiliated institutions and the research environment. The outcome was the number of peer-reviewed publications. Multivariable logistic regression analyses examined the association between the outcome and the following five research environmental factors (i.e., country of the respondents, availability of secured time for research activities or research supporting staff for the hospital, practice at a university-affiliated hospital, and years of clinical practice of 10 years or longer). Four hundred ninety responded (overall response rate: 5.6%) to the survey between June 2019 and January 2020. Fifty-five percent worked for a university-affiliated hospital, while 35% worked for a community hospital. Twenty-four percent had secured time for research within their full-time work hours. The multivariable logistic model found that a secured time for the research activities [odds ratio (OR): 2.77; 95% confidence interval (CI), 1.46–5.24], practicing at a university-affiliated hospital (OR: 2.61; 95% CI, 1.19–5.74), having clinical experience of 10 years or longer (OR:11.2; 95%CI, 1.41–88.5), and working in South Korea (OR: 2.18; 95% CI, 1.09–4.34, Reference: Japan) were significantly associated with higher research productivity. Intensivists in the three countries had limited support for their research work. Dedicated time for research was positively associated with the number of research publications.

## Introduction

The advancement of clinical medicine in Asian countries has led to an improvement in mortality among patients requiring intensive care (1–4). However, recognition in academia of critical care medicine lags behind that of North American or European countries. The growth in the number of peer-reviewed research papers from Asian countries has been sluggish except for some countries like China (5–7). Specifically, the number of accepted papers per year in high-impact critical care journals in high-income Asian countries such as Japan, South Korea, and Singapore has even remained low (Supplementary Figure 1).

There are many potential challenges to conducting high-quality studies and trials in critical care medicine due to high disease acuity and severity and heterogeneity of disease etiology of the patients, and poor resource availability, particularly in intensive care units (ICUs) in community hospitals (8–12). It has been suggested that the development of support and its system for research is central to overcome those difficulties. Trial groups such as the Canadian Critical Care Trials Group (CCCTG), for example, have established their research supporting environments such as meetings, ethics committee, a mentoring system, funding system, and research coordinators, which enabled them to conduct numerous high-quality clinical trials (13). In contrast, financial and personal support for research activities are major barriers for critical care research in lower-middle income countries (14). In addition, a survey targeting Japanese and Korean physicians identified a lack of personnel support and time for research as the main hindrance to conducting clinical trials (15).

To our knowledge, there is no study about the current research environment in critical care medicine in Asia. We conducted a cross-sectional survey targeting intensivists in Japan, South Korea, and Singapore. The objectives of this survey were to describe the current status of the research environment in the three countries and explore the differences between the countries, which can be applied for future building or improvement of the research program.

## Materials and methods

This study was a cross-sectional questionnaire survey in Japan, South Korea, and Singapore between July 2019 and January 2020.

### Eligibility criteria

We included all the physician members of the Japanese Society of Intensive Care Medicine (JSICM), the Korean Society of Critical Care Medicine (KSCCM) as of June 2019, and the Society of Intensive Care Medicine (SICM) in Singapore as of November 2019.

### Measurements

The questionnaire included four domains:

1) Characteristics of intensivists including their sub-specialty, experience of the research activity, and postdoctoral education(s).
2) Details of the work environment to conduct research.
3) Financial support environment such as grant funding opportunity.
4) Ethics committees and their activity.

### Validation of the questionnaire

We first performed face validation and content validation when creating the original questionnaire in Japanese. In the face validation, we asked all the co-investigators as content experts to evaluate whether the questionnaire measured what we intended to measure. In the content validity, we invited three critical care content experts (other than the co-investigators) from the pediatric and adult field, who individually assessed whether the questionnaire content accurately assessed all fundamental aspects of the topic. We asked them to fill out a sensibility-testing tool (Appendix 1) and a table of specifications (Appendix 2) to measure the survey's sensibility and specificity. We then modified the survey content according to the testing and the sensibility testing results.

We then translated the original Japanese version to Korean, in which the principal investigators (YK and AK) used a computer-based translation tool to prepare a draft version of the translated questionnaire and a co-investigator (SN) added grammatical corrections to it; then, a co-investigator (JHJ), who was fluent in both Japanese and Korean languages, validated it accordingly. We also translated the original Japanese version to English (AK and YK), in which JP performed final validation independently. We did not use backward translations for both languages considering that the questionnaire consisted only of closed questions without any open-ended or qualitative questions.

### Survey distribution

We collected responses using REDCap electronic data capture tools hosted at the University of Alberta (16, 17). We distributed the final survey link to the Japanese cohort's eligible physicians, obtaining individual email addresses of JSICM physician members. The JSICM physician members' email addresses were entered into the REDCap® system by an independent administrator from the JSICM office. For the Korean cohort, we distributed the final survey link in the same way as the Japanese cohort, except that the person who entered the physicians' email addresses into the REDCap system was a principal investigator (YK). The email addresses were provided and used only for the distribution to assure anonymity and privacy. For the Japanese and Korean cohorts, we sent three reminders at least 2 weeks apart (i.e., four times in total). In Singapore, we could reach all the intensivists directly by sending emails attached with URLs of the survey distributed by ICU directors in each unit. We did not send a reminder due to the time-sensitivity and the good response rate after the first invitation in Singapore.

### Statistical analyses

Descriptive data were expressed with frequencies (%). The denominator of each variable was the number of respondents answering each question. Each continuous variable's distribution is described by medians and interquartile ranges (IQRs). Mann-Whitney U test, Chi-Square test, and Fisher's exact test were used to compare the continuous and nominal variables, respectively. The Kruskal-Wallis test was used to compare continuous variables among more than two groups. We did not impute the missing data. We compared the findings of physician and ICU demographics by countries of the respondents and types of hospitals they work for, i.e., university-affiliated hospitals vs. community hospitals. We also explored the factors which could be associated with the number of peer-review papers in English by applying multivariable logistic regressions adjusted with the following five research environmental factors (i.e., country of the respondents, availability of secured time for research activities, availability of research supporting staff for the hospital, practicing at a university-affiliated hospital, and years of clinical practice of 10 years or longer). Since we could assume that the intensivists working for a university-affiliated hospital were more likely to have secured time for research activities and availability of research supporting staff, we examined the models with interaction terms for the following two factors (working for a university-affiliated hospital for secured research time and availability of research supporting personnel). A two-sided *p*-value of < 0.05 was considered statistically significant. All statistical analyses will be conducted with EZR (Saitama Medical Center, Jichi Medical University, ver. 1.36), a graphical user interface for R (The R Foundation for Statistical Computing, Vienna, Austria). The ethics committee of Kameda Medical Center and the Clinical Trial Group committee of JSICM approved this study (No. 18-021 and No. 2018004, respectively).

## Results

### Baseline characteristics of the respondents

Table 1 shows the demographic characteristics of the respondents. Among 8,824 physician members approached in each cohort, 490 responded (response rate: 5.6%) to the survey, and all the responses were included in the analyses. The specific response rate was 4.5% (323/7,106) in Japan, 7.0% (111/1,579) in South Korea, and 40.2% (56/139) in Singapore, respectively. Fifty-five percent of respondents worked for a university-affiliated hospital and 35% for a community hospital, in which the proportion of the respondents from community hospitals was higher in Japan (44%) than in the other two countries. On top of the ICU practice, nearly half of respondents practiced in the non-intensive care field by >50% such as outpatient clinics, particularly for the cohorts in Japan and South Korea. Over 80% of respondents had their clinical career of 10 years or longer. The number of ICU beds was 11 or more in 89% in South Korea, 76% in Singapore, and 53% in Japan. The number of full-time intensivists was ≤ 5 in 68%.

**Table 1:** Demographic characteristics of the respondents and their hospitals.

**Variables**	**All** **(*N =* 490)**	**Japan** **(*N =* 323)**	**South Korea** **(*N =* 111)**	**Singapore** **(*N =* 56)**
University faculty position (%)	46.1 (205/445)	43.4 (121/279)	60.9 (67/110)	30.4 (17/39)
**Location for clinical practice (%)**				
University-affiliated hospital	55.3 (271/490)	42.1 (136/323)	85.6 (95/111)	71.4 (40/56)
Community hospital	35.3 (173/490)	44.0 (142/323)	13.5 (15/111)	28.6 (16/56)
No clinical practice	9.4 (46/490)	13.9 (45/323)	0.9 (1/111)	0
Graduate student (%)	13.3 (32/240)	13.3 (21/158)	20.9 (9/43)	5.1 (2/39)
**Proportion of time for intensive care to the time of the overall clinical practice (%)**				
≥90%	21.2 (94/444)	21.9 (61/278)	26.4 (29/110)	7.1 (4/56)
50 < and <90%	30.0 (133/444)	30.6 (85/278)	30.0 (33/110)	26.8 (15/56)
0% < and <50%	38.7 (172/444)	34.5 (96/278)	35.5 (39/110)	66.1 (37/56)
None	10.1 (45/444)	12.9 (36/278)	8.2 (9/110)	0
**Proportion of the number of pediatric patients under 15 years of age among the total number of ICU patients (%)**				
100%	3.8 (15/396)	5.0 (12/240)	3.0 (3/101)	0
50 = < and <100%	3.5 (14/396)	3.3 (8/240)	5.9 (6/101)	0
0% < and <50%	46.5 (184/396)	62.5 (150/240)	26.7 (27/101)	12.7 (7/55)
None	46.2 (183/396)	29.2 (70/240)	64.4 (65/101)	87.3 (48/55)
**Specialty other than intensive care (%)**				
Anesthesia	34.1 (104/305)	37.6 (69/181)	11.1 (8/72)	53.8 (28/52)
Emergency medicine	30.8 (94/305)	45.3 (82/181)	13.9 (10/72)	3.8 (2/52)
Respiratory medicine	21.3 (65/305)	9.9 (18/181)	43.1 (31/72)	30.8 (16/52)
General medicine	4.3 (13/305)	0.6 (1/181)	8.3 (6/72)	11.5 (6/52)
Surgery (excluding cardiac surgery)	3.6 (11/305)	3.3 (6/181)	6.9 (5/72)	0
Cardiac surgery	2.6 (8/305)	2.2 (4/181)	5.6 (4/72)	0
General pediatrics (excluding pediatric intensive care/emergency medicine)	3.3 (10/305)	1.1 (2/181)	11.1 (8/72)	0
Other	0	0	0	0
**Years of clinical practice (%)**				
≥20	41.0 (182/444)	42.2 (118/278)	40.0 (44/110)	35.7 (20/56)
10–19	41.9 (186/444)	37.8 (105/278)	45.5 (50/110)	55.4 (31/56)
6–9	14.4 (64/444)	15.5 (43/278)	14.5 (16/110)	8.9 (5/56)
2–5	2.7 (12/444)	4.3 (12/278)	0	0
<2	0	0	0	0
Board-certified intensivist (%)	71.6 (318/444)	57.6 (160/278)	97.3 (107/110)	91.1 (51/56)
**Number of funded beds in ICU (%)**				
≥21	20.1 (77/384)	12.4 (29/233)	39.2 (38/97)	18.5 (10/54)
11–20	45.3 (174/384)	40.8 (95/233)	49.5 (48/97)	57.4 (31/54)
6–10	30.2 (116/384)	41.6 (97/233)	9.3 (9/97)	18.5 (10/54)
1–5	4.4 (17/384)	5.2 (12/233)	2.1 (2/97)	5.6 (3/54)
**Number of intensivists with full time employment (%)**				
≥11	13.3 (51/383)	16.4 (38/232)	0	24.1 (13/54)
6–10	18.8 (72/383)	25.0 (58/232)	4.1 (4/97)	18.5 (10/54)
1–5	56.7 (217/383)	51.3 (119/232)	87.6 (85/97)	24.1 (13/54)
None	11.2 (43/383)	7.3 (17/232)	8.2 (8/97)	33.3 (18/54)

### Factors related to research environment and the comparisons between the countries and the types of hospitals

Table 2 describes factors potentially representing the quality of the research environment for intensivists. The Singapore respondents had better full-text accessibilities to the major peer-review scientific journals than those in Japan or South Korea. Forty percent of the respondents in South Korea had secured time for research activities in which more than 50% had 5 h or more hours. A higher proportion of Korean respondents received competitive research funding (34.7% in South Korea vs. 29.1% in Japan or 16.7% in Singapore, respectively). More than half of the respondents in South Korea and Singapore had research supporting personnel in their hospital compared with 21% in Japan. Concerning the way to submit to the research ethics committee or institutional review board, a significantly lower proportion of the respondents reported the availability of online submission in Japan (36.5%) compared to the other two countries (66.7% in South Korea and 90.2% in Singapore).

**Table 2:** Factors related to research environment of the respondents.

**Variables**	**All** **(*N =* 490)**	**Japan** **(*N =* 323)**	**South Korea** **(*N =* 111)**	**Singapore** **(*N =* 56)**
**Full-text availability in the library of the hospital (%)**				
New England Journal of Medicine	67.3 (330/490)	60.1 (194/323)	76.6 (85/111)	91.1 (51/56)
Lancet	61.6 (302/490)	55.4 (179/323)	69.4 (77/111)	82.1 (46/56)
JAMA	61.8 (303/490)	55.4 (179/323)	70.3 (78/111)	82.1 (46/56)
Intensive Care Medicine	53.3 (261/490)	45.5 (147/323)	63.1 (70/111)	78.6 (44/56)
Critical Care Medicine	56.9 (279/490)	49.5 (160/323)	70.3 (78/111)	73.2 (41/56)
American Journal of Respiratory and Critical Care Medicine	42.7 (209/490)	32.2 (104/323)	59.5 (66/111)	69.6 (39/56)
**Secured time for research activities per week at the hospital (%)**				
Yes	24.3 (90/371)	20.7 (46/222)	40.0 (38/95)	11.1 (6/54)
≥20 h	2.2 (8/371)	1.4 (3/222)	4.2 (4/95)	1.8 (1/56)
10 h = < and <20 h	6.7 (25/371)	5.9 (13/222)	9.1 (10/110)	3.6 (2/56)
5 h = < and <10 h	12.4 (46/371)	10.8 (24/222)	18.2 (20/110)	3.6 (2/56)
0 < and <5 h	3.0 (11/371)	2.7 (6/222)	3.6 (4/110)	1.8 (1/56)
No	75.7 (281/371)	79.3 (176/222)	60.0 (57/95)	88.9 (48/54)
Competitive research funding as a principal investigator over the past 5 years (%)	28.7 (106/369)	29.1 (64/220)	34.7 (33/95)	16.7 (9/54)
Non-competitive research funding for the ICU (%)	14.8 (55/371)	19.4 (43/222)	7.4 (7/95)	9.3 (5/54)
**Research supporting personnel for the hospital (%)**	34.3 (127/370)	21.6 (48/222)	52.1 (49/94)	55.6 (30/54)
Epidemiologist	13.8 (51/370)	8.6 (19/222)	27.7 (26/94)	11.1 (6/54)
Biostatistician	21.6 (80/370)	13.5 (30/222)	35.1 (33/94)	31.5 (17/54)
Native English proofreader	5.7 (21/370)	2.7 (6/222)	12.8 (12/94)	5.6 (3/54)
Research assistant	11.4 (42/370)	5.9 (13/222)	18.1 (17/94)	22.2 (12/54)
Research coordinator	12.2 (45/370)	5.9 (13/222)	18.1 (17/94)	27.8 (15/54)
Other	0.5 (2/370)	0	1.1 (1/94)	1.9 (1/54)
Research supporting personnel dedicated for the ICU (%)	2.2 (8/370)	2.3 (5/222)	1.1 (1/94)	3.7 (2/54)
Access to a research ethics committee/institutional review board (IRB) at the hospital (%)	95.9 (354/369)	97.7 (216/221)	92.6 (87/94)	94.4 (51/54)
**Frequency of research ethics committee/IRB (%)**				
Regularly > once in a week	4.2 (15/354)	1.4 (3/216)	11.5 (10/87)	3.9 (2/51)
Regularly once in a month = < and < once in a week	45.5 (161/354)	52.8 (114/216)	36.8 (32/87)	29.4 (15/51)
Regularly < once in a month	18.6 (66/354)	19.0 (41/216)	17.2 (15/87)	19.6 (10/51)
Held only when requested	11.9 (42/354)	12.0 (26/216)	18.4 (16/87)	0
Don't know	19.8 (70/354)	14.8 (32/216)	16.1 (14/87)	47.1 (24/51)
**How to submit for the research ethics committee/IRB in the hospital (%)**				
Online	51.7 (183/354)	36.5 (79/216)	66.7 (58/87)	90.2 (46/51)
Not online (in any media)	35.9 (127/354)	51.4 (11/216)	18.4 (16/87)	0
Don't know	12.4 (44/354)	12.0 (26/216)	14.9 (13/87)	9.8 (5/51)

Compared with the respondents working for university-affiliated hospitals, the respondents working for community hospitals had less secured time for research activities (14.2 vs. 30.4%), less competitive and non-competitive research funds (12.1 vs. 39.0% and 4.3 vs. 21.3%, respectively), less research support personnel (21.3 vs. 42.4%), and less frequency of research ethics committee (Supplementary Table 1).

### Research productivity and the associated factors

Almost all the respondents in South Korea had at least one original publication in any language. A higher proportion of South Korean respondents had more than 20 or more than ten original English language publications than the respondents in Japan or Singapore (Table 3).

**Table 3:** Research productivity.

**Variables**	**All** **(*N =* 490)**	**Japan** **(*N =* 323)**	**South Korea** **(*N =* 111)**	**Singapore** **(*N =* 56)**
Original article* in any language as the first or corresponding author (%)	77.0 (341/443)	72.6 (201/277)	95.6 (105/110)	62.5 (35/56)
Number of original articles*published in English as the first or corresponding author (%)				
≥21	11.5 (39/339)	8.5 (17/200)	20.2 (21/104)	2.9 (1/35)
11–20	11.5 (39/339)	9.0 (18/200)	17.3 (18/104)	8.6 (3/35)
6–10	14.5 (49/339)	11.0 (22/200)	21.2 (22/104)	14.3 (5/35)
1–5	53.4 (181/339)	56.5 (113/200)	40.4 (42/104)	74.3 (26/35)
0	9.1 (31/339)	15.0 (30/216)	1.0 (1/104)	0

^*^Case reports or letters to editors are excluded.

Table 4 shows the multivariable logistic regression analysis result to examine the potential factors associated with the number of peer-review publications. The adjusted odds ratio (OR) to have more than ten original peer-reviewed publications in English for those with a secured time for the research activities was 2.77 (95% confidence interval (CI), 1.46–5.24, *p* = 0.002) compared to the respondents without. The adjusted OR was 2.61 in the respondents practicing at a university-affiliated hospital (95%CI, 1.19–5.74, *p* = 0.017) compared to the others. The adjusted OR was 11.2 when having clinical experience of 10 years or longer (95%CI, 1.41–88.5, *p* = 0.022), compared to those with clinical experience of <10 years. When comparing the cohorts by countries, the odds ratio was significantly higher for the Korean cohort (OR: 2.2; 95%CI, 1.1–4.3) compared to it in Japan. The models including an interaction term did not present significant evidence of interactions. When we looked at the respondents who worked for a university-affiliated hospital with secured time for research activities and the research supporting staff in their hospital, the odds to have more than ten English publications was 2.4 (95%CI, 1.1–5.2) compared to those without all the three factors.

**Table 4:** Associated factors with the number of publications.

**Independent variables**	**The number of publications**			**Adjusted**		
	**≧11**	**10≦**	***p*-value**	**Odds Ratios**	**95% CIs**	***p*-value**
**Country**			<0.001			
Japan	44.9% (35/78)	63.2% (165/261)		Reference		
South Korea	50.0% (39/78)	24.9% (65/261)		2.18	1.09–4.34	0.027
Singapore	5.1% (4/78)	11.9% (31/261)		0.50	0.15–1.64	0.25
Secured time for research activities (Yes/No)	51.5% (35/68)	22.7% (50/220)	<0.001	2.77	1.46–5.24	0.002
Research supporting personnel for the hospital (Yes/No)	45.3% (29/64)	37.5% (78/208)	0.31	0.80	0.41–1.55	0.50
Practice at a university–affiliated hospital (Yes/No)	80.8% (63/78)	63.2% (165/261)	0.004	2.61	1.19–5.74	0.017
Years of clinical practice ≧10 years (Yes/No)	97.4% (76/78)	87.7% (229/261)	0.010	11.2	1.41–88.5	0.022

95% CI, 95% confidence interval.

## Discussion

This study is the first report describing the current status of the research environment for intensivists in the three Asian countries. There were some differences in the baseline characteristics of the respondents between the three countries, such as type of hospital/ICU they worked for and years of clinical experience. However, we found a commonality such that a large proportion of the intensivists lacked secured time for the research activities, research supporting personnel, and research funding in their hospital. Also, there were substantial differences in the secured time for the research and research supporting staff in the hospital among the three countries.

Our results were consistent with previous studies evaluating challenges for critical care research. A scoping review in lower-middle income settings identified limited funding/investment, inadequate access to mentors/training, and limited research/statistical support staff as barriers in critical care research in lower-middle income countries (14). An international survey on barriers and facilitators of conducting randomized controlled trials in pediatric critical care reported that lack of funding was the major barrier and that protected time for research, a stable recruit system, collaboration with a research network, government funding, and academic department support were the facilitators (18). Recently, international critical care randomized trials have overcome these challenges through sharing the resources and published important papers incorporating areas with inadequate research infrastructure in the context of the pandemic of coronavirus disease 2019 (19, 20).

Based on our results, one way to overcome the barriers can be to build a collaborative environment between academics and non-academics such as mentoring or sharing research resources. Our study suggests that this is particularly important for the intensivists working for a community hospital supposed to have limited research support or resources, shown in prior literature (12, 15, 21). These collaborations would help build a robust and sustainable clinical research program in community hospitals (11). We should also acknowledge the potential benefits for the researchers in academic sites in constructing these collaborations by increasing the number and representativeness of the patients enrolled, which is crucial given the heterogeneity in patients' characteristics (8, 22). Given the complexity of the contemporary study methodologies, supports from experienced personnel such as epidemiological, statistical analysis, and data management are also essential for all intensive care researchers to perform high-quality clinical research.

Building a financial support system for the researchers in community hospitals should be another essential aspect of promoting research. For example, in Japan and South Korea, competitive government-based research funds ask applicants to belong to an academic institution, which has been a significant challenge for the researchers working in non-academic institutions (23). Besides, out-of-hours work related to research activities is often done without extra remunerations, which may lead to a great shackle about the continuity of researchers' motivation (21).

The limited resources of critical care practice in Asia could also contribute to its poor research productivity. A lack of organizational and human resources for ICUs is a major problem worldwide (24). Of note, according to a global survey concerning ICU structure, the ICUs in Asia were managed in open formats more frequently than those in Europe or Oceania. In addition, the Asian ICU had fewer admissions per year compared with those in North America or Oceania (25). Open ICUs might limit consistent, intensive care delivery or systematic data collection, and fewer ICU admissions would prevent timely patient recruitment. Since previous meta-analyses showed that closed ICUs have better patient outcomes (26) than open ones and that critically ill patients were more likely to benefit from high-volume centers than low-volume ones (27), aggregating small ICUs into a larger one could improve both critical care practice and research in Asia (23).

In this study, we found that the intensivists in Korea had more peer-reviewed publications and better research supporting infrastructure than those in the other two countries. This result may be because a higher proportion of Korean respondents worked for academia. Although this survey was emailed to all the KSCCM members, most Korean respondents worked for university-affiliated hospitals. It may come from a selection bias, which means those who are not interested in research activities did not respond to the survey. This trend was a little stronger in South Korea because responding to the survey was promoted to the board of committee members of the KSCCM.

Ethical issues can be a challenge for critical care research given the urgency of patient condition and the difficulty of obtaining informed consent from the patient (8). Applying an alternative method of informed consent, such as deferred consent, could help us enroll patients; however, institutions and institutional review boards (IRBs) that can understand and practice those methods are still limited. It will be necessary to share the know-how among the institutions and promote such as central IRBs to break down the barriers (13).

Lastly, to further boost critical care science, it is essential to train new intensivists to pursue research. Providing a roadmap in the research career will attract them and alleviate worries for their future job security. A survey conducted in the United States reported that most clinical trainees felt that spending time and research activity efforts would not necessarily lead to their job security or even a better job (28, 29). It should be necessary to build a program to nurture new researchers in Asia across countries and societies.

There are limitations to this study:

1. Due to the nature of cross-sectional research, a thorough causal inference could not be made in this study's findings.
2. A selection bias could have occurred. For instance, intensivists interested in research activities might have responded more to this survey. Besides, because most KSCCM members worked for university-affiliated hospitals, working for South Korea and working for a university-affiliated hospital can be confounding.
3. The overall response rate was low. The denominators used for the rate calculation in the Japanese cohort included specific proportions of physicians who were not actively practicing in intensive care such as anesthesiology or emergency medicine. We applied a rigorous methodology in the development and administration stage of this survey to minimize the biases.

In conclusion, intensivists in the three high-income Asian countries had limited research supporting environment and infrastructure with some variations among the countries. It is necessary to build a collaborative environment between academia and non-academia and across countries to share the experience and resources, and to present various roadmaps to new intensive care researchers.

## Data availability statement

The raw data supporting the conclusions of this article will be made available by the authors, without undue reservation.

## Ethics statement

The studies involving human participants were reviewed and approved by the Ethics Committee of Kameda Medical Center. Written informed consent for participation was not required for this study in accordance with the national legislation and the institutional requirements.

## Author contributions

YK conceptualized and designed the study, built a survey database, conducted data collection and cleaned the data, carried out analyses, drafted the initial article, revised the initial article, and approved the final article as submitted. AK conceptualized and designed the study, drafted the initial article, revised the initial article, and approved the final article as submitted. NS, TK, and HY conducted a pre-testing, contributed in designing the survey, revised the initial article, and approved the final article as submitted. NS, JP, and JJ played a role in leading and organizing survey distribution in each country, contributed in designing the survey, revised the initial article, and approved the final article as submitted. All authors approved the final article as submitted and agree to be accountable for all aspects of the work.

## Conflict of interest

The authors declare that the research was conducted in the absence of any commercial or financial relationships that could be construed as a potential conflict of interest.

## Publisher's note

All claims expressed in this article are solely those of the authors and do not necessarily represent those of their affiliated organizations, or those of the publisher, the editors and the reviewers. Any product that may be evaluated in this article, or claim that may be made by its manufacturer, is not guaranteed or endorsed by the publisher.
