# Supplementary material for: The research environment of critical care in three Asian countries: A cross-sectional questionnaire survey
Source: Front Med (Lausanne). 2022 Sep 20;9:975750. doi: 10.3389/fmed.2022.975750 (PMC9530362; doi:10.3389/fmed.2022.975750)
Supplement: Supplementary file 1 [file Table_1.pdf]

Supplemental Table 1 Comparison of factors related to research environment of the respondents between university-affiliated and community hospitals.

Variables	University-affiliated hospitals (N = 271)	Community hospitals (N = 173)
Full-text availability in the library of the hospital (%)		
New England Journal of Medicine	78.2 (212/271)	68.2 (118/173)
Lancet	73.1 (198/271)	60.1 (104/173)
JAMA	72.7 (197/271)	61.3 (106/173)
Intensive Care Medicine	68.6 (186/271)	43.4 (75/173)
Critical Care Medicine	70.1 (190/271)	51.4 (89/173)
American Journal of Respiratory and Critical Care Medicine	59.8 (162/271)	27.2 (47/173)
Secured time for research activities per week at the hospital (%)		
Yes	30.4 (70/230)	14.2 (20/141)
>=20 hours	2.6 (6/230)	1.4 (2/141)
10 hours=< and <20 hours	8.7 (20/230)	3.5 (5/141)
5 hours=< and <10 hours	15.7 (36/230)	7.1 (10/141)
0< and <5 hours	3.5 (8/230)	2.1 (3/141)
No	69.6 (160/230)	85.8 (121/141)
Competitive research funding as a principal investigator over the past five years (%)	39.0 (89/228)	12.1 (17/141)
Non-competitive research funding for the ICU (%)	21.3 (49/230)	4.3 (6/141)
Research supporting personnel for the hospital (%)	42.4 (97/229)	21.3 (30/141)
Epidemiologist	14.0 (38/271)	7.5 (13/173)
Biostatistician	21.0 (57/271)	13.3 (23/173)
Native English proofreader	4.4 (12/271)	5.2 (9/173)

Research assistant	11.1 (30/271)	6.9 (12/173)
Research coordinator	11.8 (32/271)	7.5 (13/173)
Other	0.4 (1/271)	0.6 (1/173)
Research supporting personnel dedicated for the ICU (%)	0.4 (1/222)	0.6 (1/173)
Access to a research ethics committee/institutional review board (IRB) at the hospital (%)	96.1 (220/229)	95.7 (87/94)
Frequency of research ethics committee/IRB (%)		
Regularly > once in a week	5.9 (13/220)	1.5 (2/134)
Regularly once in a month =< and <once in a week	50.9 (112/220)	36.6 (49/134)
Regularly <once in a month	14.5 (32/220)	25.4 (34/134)
Held only when requested	8.2 (18/220)	17.9 (24/134)
Don't know	20.5 (45/220)	18.7 (25/134)
How to submit for the research ethics committee/IRB in the hospital (%)		
Online	64.5 (142/220)	30.6 (41/134)
Not online (in any media)	24.5 (54/220)	54.5 (73/134)
Don't know	10.9 (24/220)	14.9 (20/134)
