# Supplementary material for: The research environment of critical care in three Asian countries: A cross-sectional questionnaire survey
Source: Front Med (Lausanne). 2022 Sep 20;9:975750. doi: 10.3389/fmed.2022.975750 (PMC9530362; doi:10.3389/fmed.2022.975750)
Supplement: Supplementary file 2 [file Data_Sheet_1.PDF]

Appendix 1. Sensibility-testing tool

研究責任者である小谷祐樹（亀田総合病院）、川口敦（アルバータ大学）より、「集中治療における研究とその環境：二カ国横断調査」の質問紙に関する臨床感度テストへのご協力のお願いです。

1. 集中治療における研究環境の重要なテーマを漏れなく質問できていますか。（丸をつけてください）

非常に<br>悪い	悪い	普通	良い	非常に<br>良い

漏れているテーマがあれば記載して下さい。 ______________________________

2. 質問に対する回答選択肢はわかりやすいですか。（丸をつけてください）

非常に<br>わかりにくい	やや<br>わかりにくい	まあまあ<br>わかりやすい	わりと<br>わかりやすい	非常に<br>わかりやすい

3. 不適切、もしくは冗長な質問はいくつありますか。（丸をつけてください）

非常に<br>多い	多い	いくらか<br>ある	少ない	非常に<br>少ない

不適切、もしくは冗長な質問を挙げてください。 ______________________________

4. この質問紙を通して、集中治療医の研究環境について、知ることができそうですか。（丸をつけてください）

可能性は<br>非常に低い	可能性は<br>低い	普通	可能性は<br>高い	可能性は<br>非常に高い

5. 質問紙の回答にかかった合計時間を記入してください。 __________分

御意見があればこの用紙の裏に記載して下さい。

臨床感度テストにご協力いただきありがとうございました。

"A guide for the design and conduct of self-administered surveys of clinicians." CMAJ 2008;179(3):245-52. より修正
