# Supplementary material for: The research environment of critical care in three Asian countries: A cross-sectional questionnaire survey
Source: Front Med (Lausanne). 2022 Sep 20;9:975750. doi: 10.3389/fmed.2022.975750 (PMC9530362; doi:10.3389/fmed.2022.975750)
Supplement: Supplementary file 3 [file Data_Sheet_2.PDF]

Appendix 2. Table of specifications

特異度表 集中治療における研究とその環境：二カ国横断調査

研究の目的・意義

集中治療に従事する医師が直面する研究に関連する環境、現状を記述することで、今後の同分野医学の発展に繋がる研究、教育基盤情報となることが期待できる。また日本と韓国の二カ国間での異差あるいは両国と他先進国からの既存情報を比較することで、問題点等をより深く検証することができる。最も当てはまるものに丸をつけて下さい。

テーマ	収集項目	重要性<br>（高、中、低）	質問・回答は適切か<br>（良い、普通、悪い）
**医師・施設属性**			
	医師の背景の差（専門分野、経験年数など）	高　中　低	良い　普通　悪い
	施設の差（病床数、症例数など）	高　中　低	良い　普通　悪い
**研究に関連した周辺環境**			
	研究活動のために確保された時間	高　中　低	良い　普通　悪い
	人的支援	高　中　低	良い　普通　悪い
	研究助成金	高　中　低	良い　普通　悪い
	指導体制	高　中　低	良い　普通　悪い
	倫理委員会	高　中　低	良い　普通　悪い
**その他の意見**			
	将来の希望	高　中　低	良い　普通　悪い
	研究環境の問題点	高　中　低	良い　普通　悪い
