# Supplementary figures and images for: The research environment of critical care in three Asian countries: A cross-sectional questionnaire survey
Source: Front Med (Lausanne). 2022 Sep 20;9:975750. doi: 10.3389/fmed.2022.975750 (PMC9530362; doi:10.3389/fmed.2022.975750)

## Number of articles

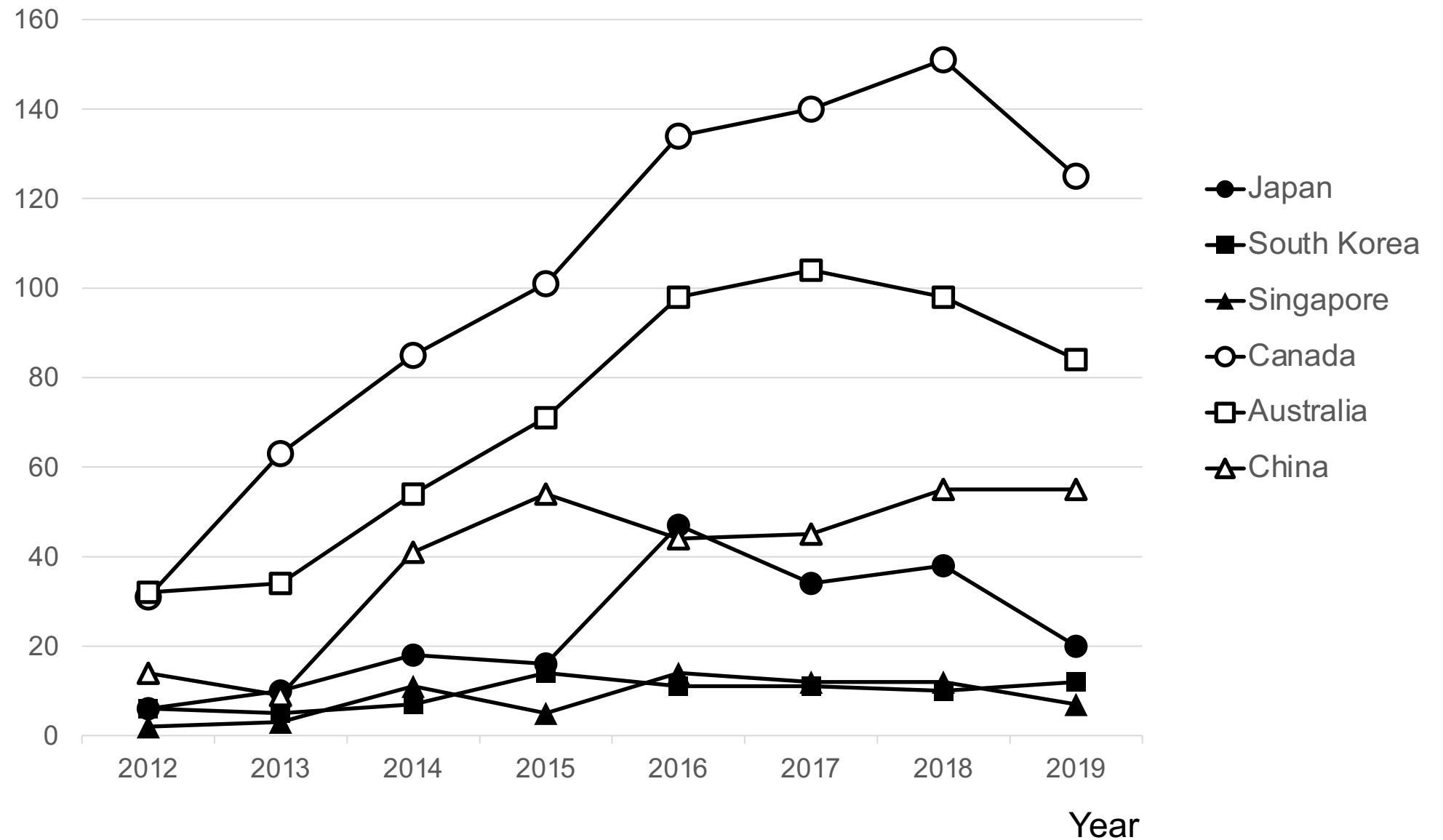

Supplement: Supplementary file 4 [file Data_Sheet_3.PDF]
